# Supplementary figures and images for: Mechanism of enterovirus VP0 maturation cleavage based on the structure of a stabilised assembly intermediate
Source: PLoS Pathog. 2024 Sep 19;20(9):e1012511. doi: 10.1371/journal.ppat.1012511 (PMC11444389; doi:10.1371/journal.ppat.1012511)

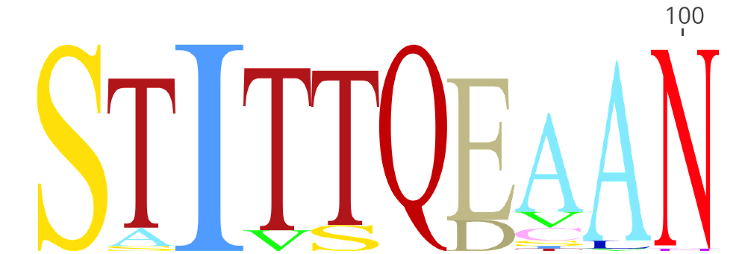

Supplement: S1 Fig — Sequence logo generated from 7955 sequences, representing all enterovirus types (EV A-L, RV A-C). Displayed VP0 amino acid range 90–100. (PNG) [file ppat.1012511.s001.png]

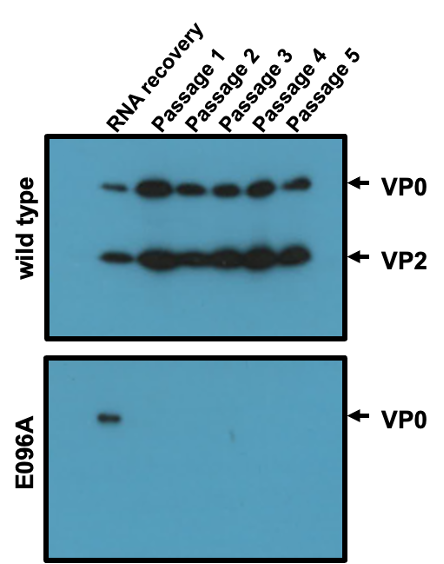

Supplement: S2 Fig — Virus recovered form WT or E096A mutant viral RNA electroporated into HeLa cells. The recovered virus was passaged through HeLa cells for a total of 5 passages. No replication was detected in E096A mutant samples determined by visual inspection for signs of CPE and assessment of EVA71 proteins by western blot. Western blots show WT EVA71 and E096A mutant EVA71 samples of cell culture supernatant probed for the presence of VP0 and VP2 using mAb 979 and an anti-mouse HRP, shown representative western blot, n = 3. (PNG) [file ppat.1012511.s002.png]

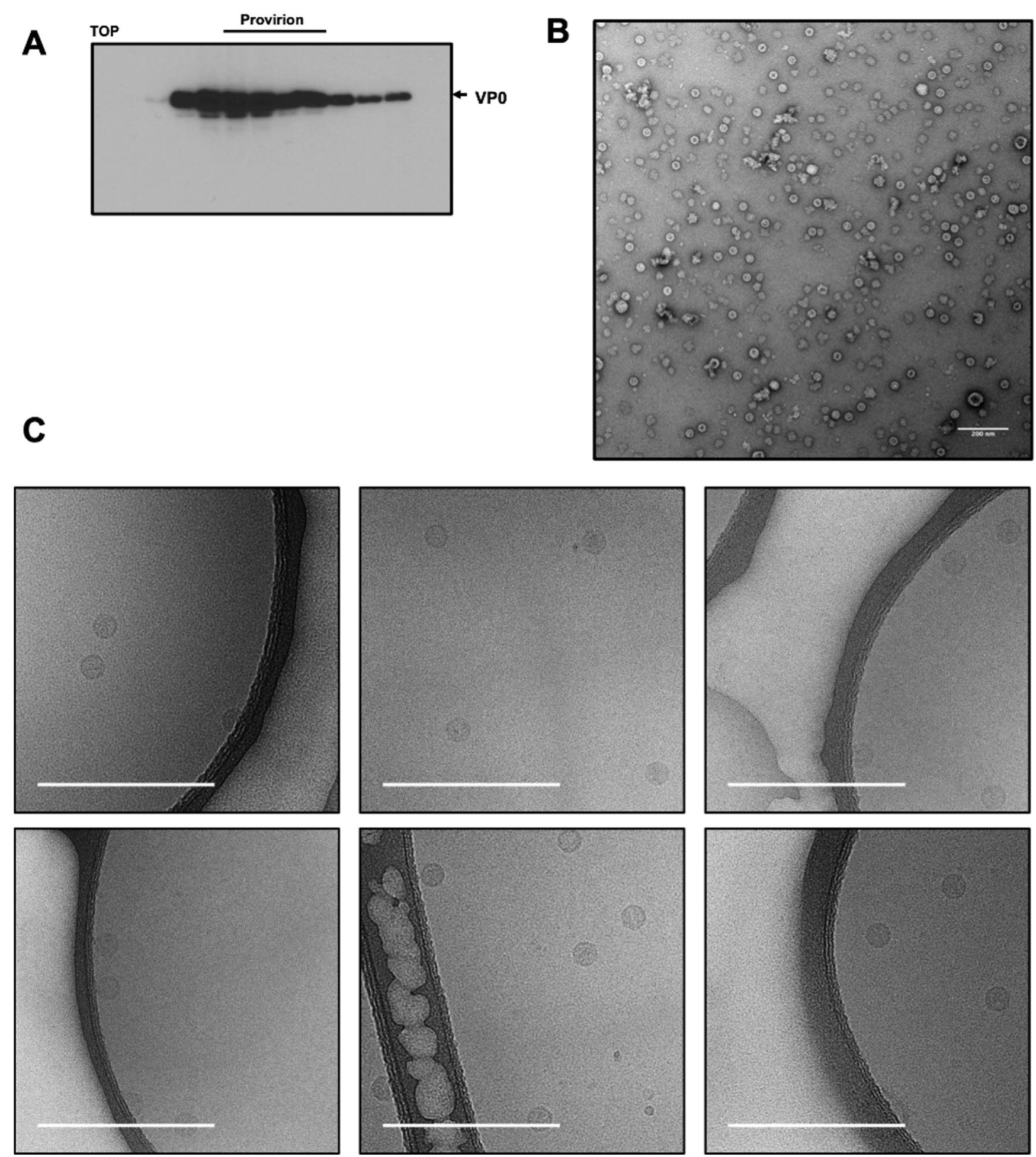

Supplement: S3 Fig — EVA71 E096A provirions were recovered directly from T7 transcribed RNA and purified through a 30% (w/v) sucrose cushion, before being separated along a 15–45% sucrose gradient. Fractions corresponding to provirions were subsequently diluted in PBS and underlaid with 25–45% sucrose and were further separated. A) Fractions were collected and assessed for the presence of VP0 and VP2 using mAb979. B) Peak fractions were then concentrated across a 100 kDa mwco spin concentrator with several PBS washes to remove excess sucrose. Concentrated samples were then viewed by TEM after being stained with 2% UA, C) Example cryoEM micrographs. Scale bars = 200 nm. (PNG) [file ppat.1012511.s003.png]

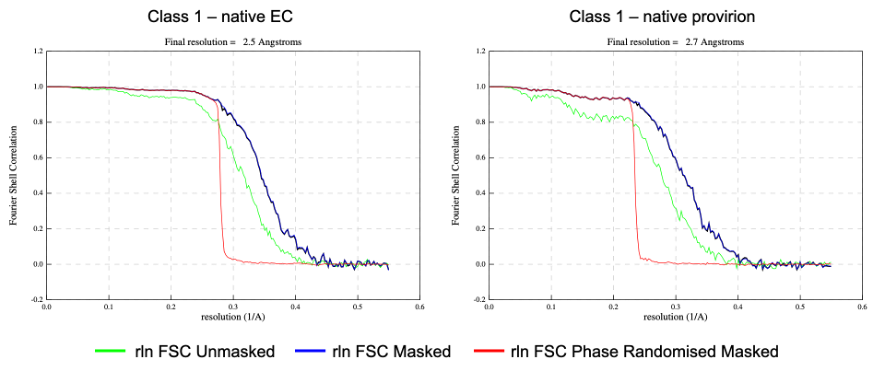

Supplement: S4 Fig — Fourier shell correlation (FSC) for native EC and native provirion maps, resolved to 2.5 Å and 2.7 Å, respectively, using the gold-standard (0.143). (PNG) [file ppat.1012511.s004.png]

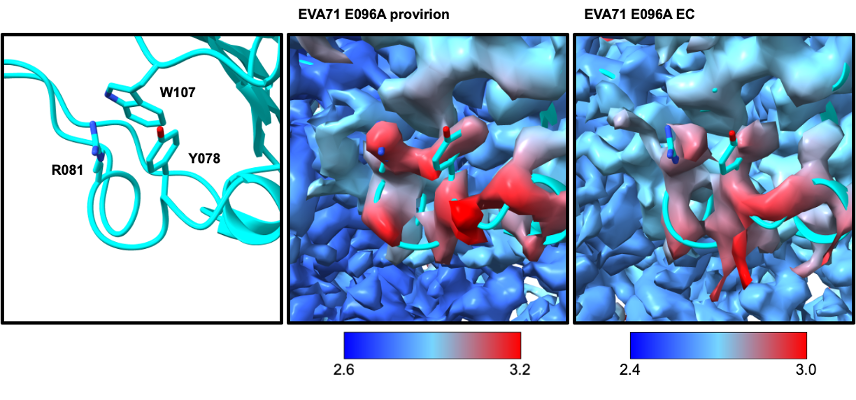

Supplement: S5 Fig — Stick diagram and local resolution rendered density map for the E096A provirion (middle) and EC (right). (PNG) [file ppat.1012511.s005.png]

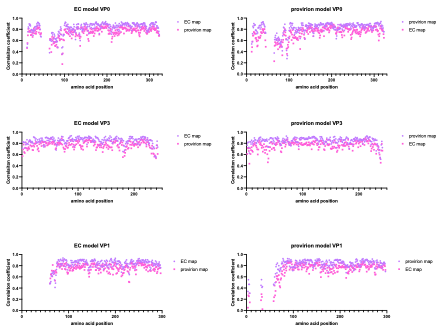

Supplement: S6 Fig — Correlation coefficients for EC modelled residues (left) fitted into the EC map (purple) or provirion map (pink), and provirion modelled residues (right) fitted into the provirion (purple) or EC (pink) map. *in both instances the cognate map/model pair are displayed in purple. (PNG) [file ppat.1012511.s006.png]

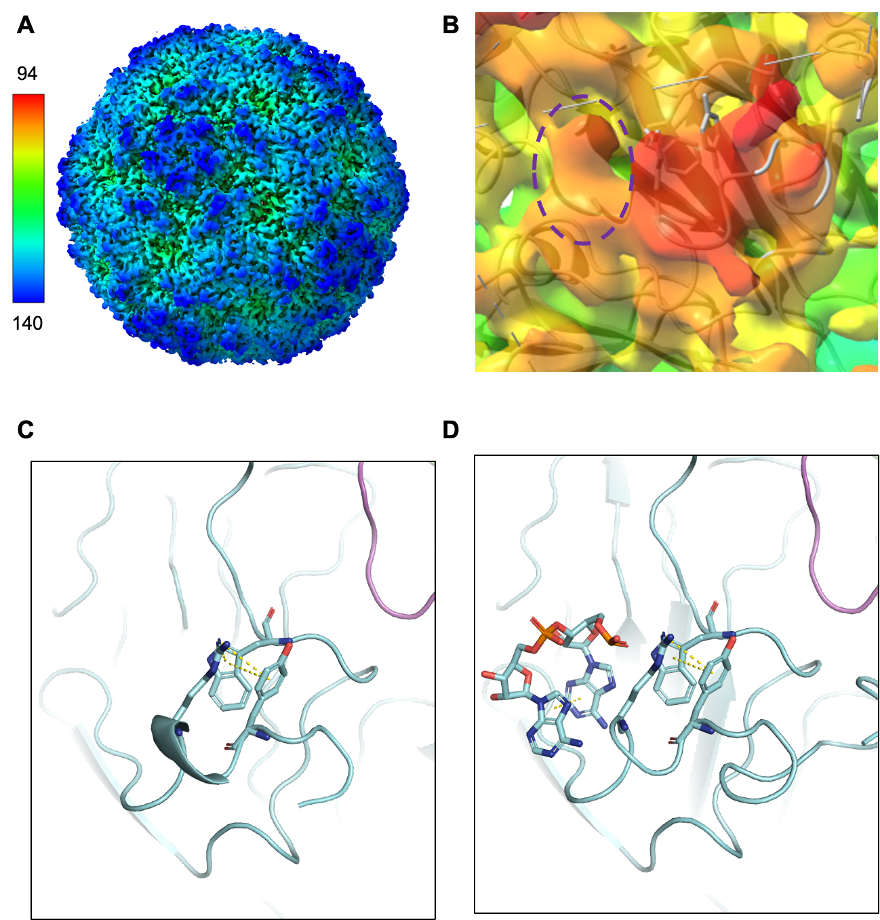

Supplement: S7 Fig — The EVA71 E096A provirion was processed through a low-pass filter to 5 Å. A) Full map after processing and B) regions of interest proximal to W107, R081, Y078. Additional region of globular density indicated in the dotted ellipse. Map coloured by radial distance in Å. C) Refined model of EVA71 E096A provirion with VP0 Y078, R081, W107 stick model and pi-interactions displayed. D) as C) but with RNA dinucleotide with pi-interactions included. (PNG) [file ppat.1012511.s007.png]

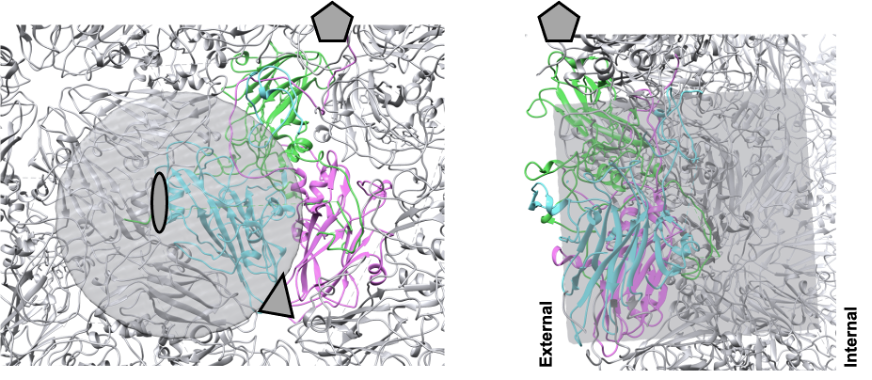

Supplement: S8 Fig — Location of the mask used for focussed classification. A cylindrical mask with a radius of 32 pixels and a depth of 64 pixels was placed over the reference asymmetric unit in order to cover regions with globular density and flexible regions. (PNG) [file ppat.1012511.s008.png]

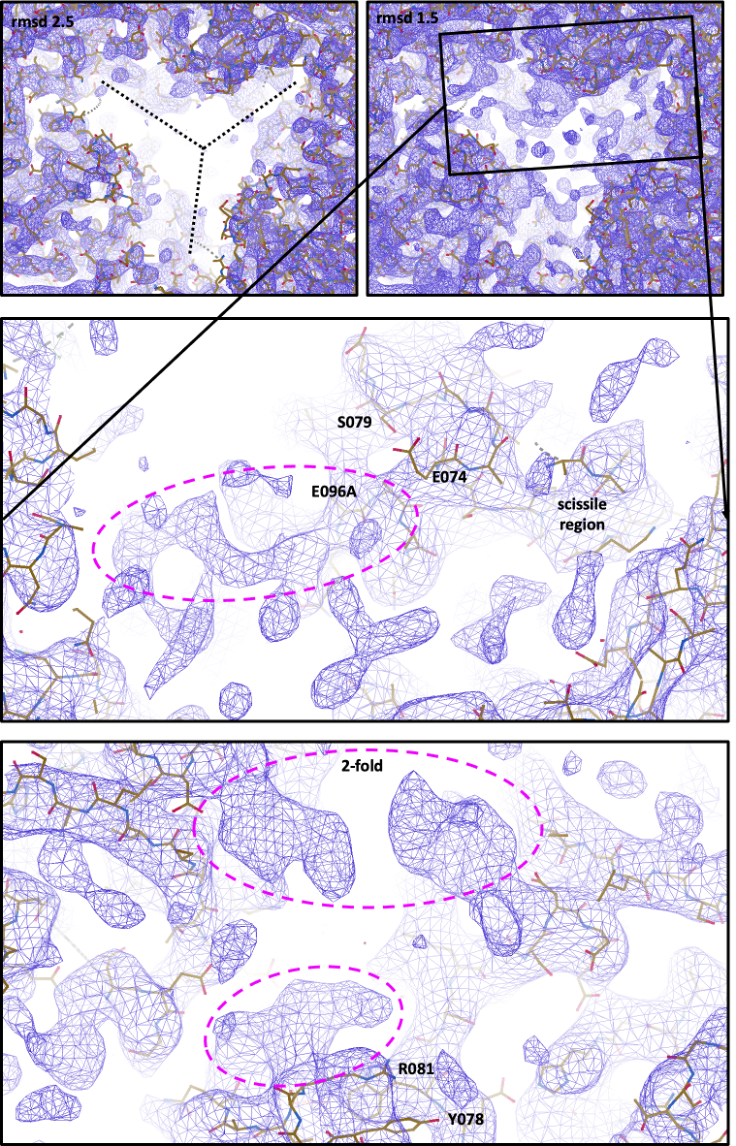

Supplement: S9 Fig — After focussed classification was performed on the EVA71 E096A mutant provirion, the resultant map was processed through a low-pass filter. The map was displayed at 2.5 rmsd (top left) and 1.5 rmsd (top right). Density was noted in proximity to the E096A mutant residue (middle), in proximity to VP0 W107, and beneath the 2-fold symmetry axis (bottom). Regions of unoccupied density are indicated by the purple dotted ellipse. (PNG) [file ppat.1012511.s009.png]

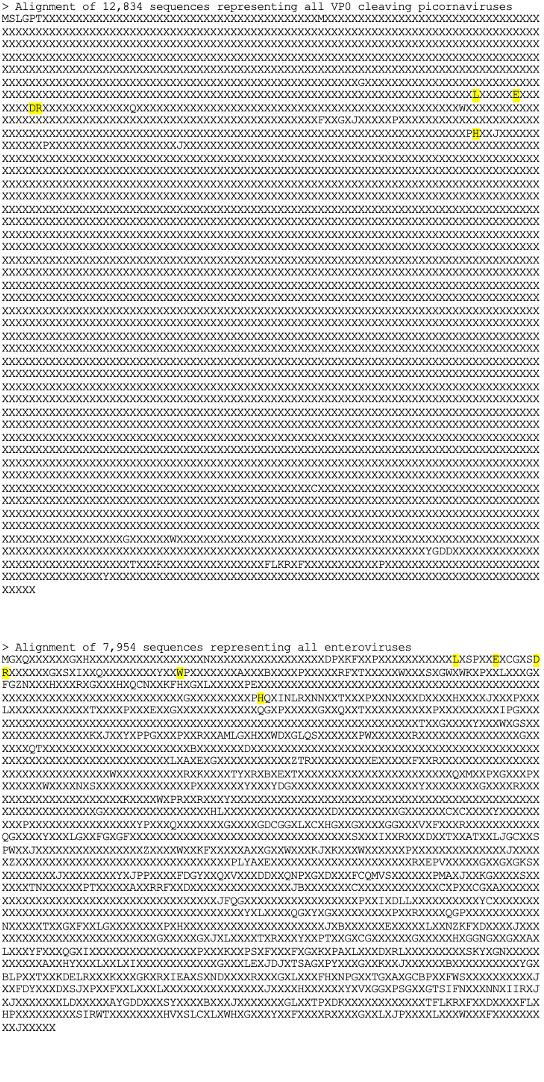

Supplement: S10 Fig — 99% consensus sequence of picornaviruses which cleave VP0 (top) and enteroviruses (bottom). Highlighted residues: 1) a Leu residue conserved in the P2 position of the scissile boundary, 2) the presence of a Glu residue at VP0 residue 74 (P5’), 3) an aromatic residue at VP0 position 78 (Tyr/Phe) (does not display as a conserved residue), 4) an Asp residue at VP0 position 80, 5) an Arg residue at VP0 position 81, 6) a negatively charged residue at VP0 position 96 (Asp/Glu), (does not display as a conserved residue), 7) Trp between the A1/2 β-sheets, 8) a His residue at near the N-terminal end of the VP0 F-strand. (PNG) [file ppat.1012511.s010.png]

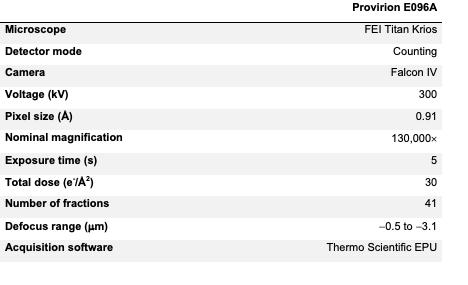

Supplement: S1 Table — (PNG) [file ppat.1012511.s011.png]

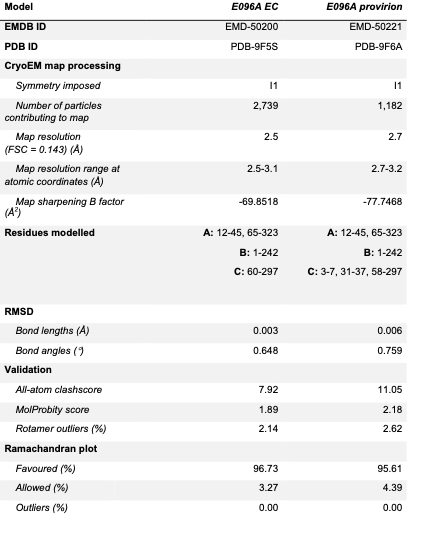

Supplement: S2 Table — (PNG) [file ppat.1012511.s012.png]
